# The two-faced role of ATF2 on cisplatin response in gastric cancer depends on p53 context

**DOI:** 10.1186/s13578-022-00802-w

**Published:** 2022-05-31

**Authors:** Lingxue Xu, Jingjing Wang, Danhua Zhang, Lijie Song, Han Wu, Jianyao Wang, Jinxin Miao, Haoran Guo, Sujuan Fang, Lingling Si, Jingfei Chen, Yifan Wu, Yangyang Wu, Lihong Wang, Na Zhang, Louisa Chard, Yaohe Wang, Zhenguo Cheng

**Affiliations:** 1 National Center for International Research in Cell and Gene Therapy, Sino-British Research Centre for Molecular Oncology, State Key Laboratory of Esophageal Cancer Prevention and Treatment, School of Basic Medical Sciences, Academy of Medical Sciences, Zhengzhou University, 450052 Zhengzhou, China; 2 Department of Surgical Oncology, The First Affiliated Hospital of Zhengzhou University, 450052 Zhengzhou, China; 3 Department of Medical Oncology, The First Affiliated Hospital of Zhengzhou University, 450052 Zhengzhou, China; 4 Centre for Biomarkers & Biotherapeutics, Barts Cancer Institute, Queen Mary University of London, E1 4NS London, United Kingdom

**Keywords:** ATF2, p53, ERK1/2, Cisplatin, Gastric cancer, Prognosis

## Abstract

**Background:**

Activating transcription factor-2 (ATF2) is a member of the basic leucine zipper family of DNA-binding proteins, which exhibits both oncogenic and tumor suppression activity in different tumors. However, the molecular mechanism of its dual function in cancer chemotherapy especially in gastric cancer has still not been elucidated.

**Methods:**

The protein expression and location of ATF2 in gastric cancer tissues was detected with immunohistochemistry assay, and the clinical significance was analyzed using TCGA and GEO database. The activation and impact of ATF2 in cisplatin treated cells were evaluated with western blot, incucyte live cell analysis, clone formation and tumor xenografts assays. Interaction between ATF2 and p53 was confirmed with immunoprecipitation and GST-pull down. Potential molecular mechanism of ATF2 in different p53 status cells was analyzed with RNA sequencing and real-time quantitative PCR.

**Results:**

ATF2 mainly located in the nucleus of cancer cells, higher ATF2 level was associated with poor five-year survival of gastric patients, especially in those undergone chemotherapy treatment. Cisplatin treatment significantly activated ATF2 in p53 mutant cells. ATF2 could interact with the trans-activation domain of p53 and enhance cisplatin sensitivity in p53 wild type cell lines, while promoted cell survival in mutant p53 cancer cells by affecting ERK1/2 pathway.

**Conclusions:**

This study confirmed the effect of ATF2 on cisplatin sensitivity was associated with the functional status of p53 in gastric cancer cells. Integrated analysis of ATF2 expression and P53 status could be used to evaluate the chemotherapy sensitivity and prognosis of gastric cancer patients.

**Supplementary information:**

The online version contains supplementary material available at 10.1186/s13578-022-00802-w.

## Background

Gastric cancer (GC) is the fifth most common malignant tumor and the third leading cause of cancer death worldwide [1]. In China, the incidence and mortality of GC is the second highest after lung cancer [2]. Although advances in surgery and radiotherapy, chemotherapy, targeted therapy as well as biotherapy have improved the clinical prognosis of patients with GC, the five-year survival rate remains less than 30% due to its high propensity to recurrence [3]. Therefore, it is of extreme importance to understand the mechanism by which therapeutic tolerance arises in GC.

ATF2 (activating transcription factor 2) is a member of the ATF and CREB groups in the basic leucine zipper (bZIP) transcription factor family [4]. It is an ubiquitously expressed protein with a particularly abundant distribution in the brain [5]. ATF2 can interact with other AP1 transcription factors (e.g. Jun, Creb, Fos) to form homo- or hetero-dimer complexes, which confer ATF2 its various cellular functions, such as embryonic development, disease development, stress response (e.g. DNA damage response), chromatin remodeling and transcription-independent mitochondrial abnormality [4, 6–9]. In response to stress stimuli or cytokine stimulation, several critical kinases (such as JNK, P38 and ERK) activate ATF2 by phosphorylating the threonine residues Thr69 and Thr71, then regulate the transcription of various downstream target genes [10–14].

Current studies suggest that ATF2 can exert oncogenic activities or tumor suppressor function depending on the tissues or cell type. It has been shown to possess carcinogenic activity in melanoma and lung cancer[15, 16], while it exerts tumor suppressor activity in non-malignant skin cancer and breast cancer [17]. Lau et al. reported that ATF2 nuclear localization was associated with its oncogenic activity, while mitochondrial localization performs tumor suppressor function via impairment of the mitochondrial action potential when cells are exposed to genotoxic stress[16], indicating that ATF2 could exert opposite functions in relation to its subcellular localization. Recent study found ATF2 participated in the endocrine treatment resistance of breast cancer by modulating ER expression and activity[18]. Although our previous study had demonstrated that ATF2 is involved in the hematogenous metastasis of GC, the role that ATF2 plays in the response of GC to chemotherapy remains unclear.

In this paper, we evaluated the effect of ATF2 on the chemo-sensitivity of GC, and elucidated the underlying mechanisms responsible which will open new avenues for GC therapy.

## Methods

### Cell culture and stable cell lines construction

GC cell lines MGC-803, HGC-27, AGS and colorectal cancer cell lines HCT-116 were purchased from the Cell Bank of the Chinese Academy of Sciences. HCT-116 (p53^−/−^) cells were provided by Professor Liu Cao at the China Medical University. All cells were cultured in proper medium supplemented with 10% fetal bovine serum (Lonza Biowhittaker), 100 U/ml penicillin and streptomycin at 37  ℃ in 5% CO_2_. ATF2-WT, ATF2-T71A, ATF2-T71E stable cell lines were constructed using lentivirus as previous described [19].

### Western blotting assay

Total cell protein was isolated with RIPA lysis buffer containing protease and phosphatase inhibitors (Roche), and protein concentration was measured using a BCA assay. 20 µg total protein was separated with 8% SDS-PAGE gel, and transferred to polyvinylidene difluoride membrane (Millipore). Membranes were blocked with 5% non-fat powder milk for one hour at room temperature, then hybridized with appropriate primary antibodies overnight at 4 ℃. Antibodies including p53 (SC-126, Santa Cruz Biotechnology, against the N terminal of p53), p-Ser15-p53 (Cell Signaling Technology), ATF2 (Santa Cruz Biotechnology), p-Thr71 ATF2 (Cell Signaling Technology), Flag tag (Abmart), GAPDH antibody (Proteintech), were used according to manufacturers’ instructions. Membranes were incubated with appropriate secondary antibodies (ZSGB Bio-technology) for one hour at room temperature and detected with enhance chemiluminescence (ECL) reagent (Thermo Pierce).

### Immunoprecipitation (IP) assay

MGC-803 cells (5 × 10^6^) were seeded in a 10 cm culture dish and co-transfected ATF2 and p53 plasmids. After six hours, medium was replaced and cells were further cultured for 36 h. For endogenous IP assay, 5 × 10^6^ MGC-803 cells were seeded and treated with 2 µg/ml cisplatin for 24 h. Cells were washed and lysed with 500 µl IP lysis buffer (containing protease inhibitors and 1mM PMSF). 5 µl flag tag antibody (or 10 µl ATF2 antibody) and 50 µl pre-washed Protein A/G magnetic beads (MedChemExpress) were added to the lysis buffer which contained 2 mg protein, and this was incubated overnight at 4 ℃. Protein-antibody-beads complex were washed three times with IP lysis buffer, and the complex harvested by centrifugation at 500 × g for 5 min. All samples were analyzed using a Western Blot assay.

### Immunofluorescence staining

1 × 10^4^ cells were seeded on sterilized glass coverslip in 12-well plates. For the drug-treatment groups, cells were cultured with cisplatin for 24 h. Cells were washed three times with PBS, fixed with 4% paraformaldehyde (PFA) for 15 min, and then washed three times with PBS for 10 min. Cells were penetrated using 0.1% Triton X-100 for 10 min, then blocked with goat serum for one hour at room temperature. Subsequently, cells were incubated with primary antibody overnight at 4 ℃, washed with PBS and detected by fluorescence conjugated secondary antibody (Invitrogen) for one hour at room temperature. Finally, cells were analyzed by a confocal microscope at the original magnification 400×.

### Cell proliferation and cytotoxicity assays

ATF2 over-expressing or control cells were seeded in 96-well plates at 3000 cells/well. 24-hour later, cisplatin was added into cells and the plates were transferred into the IncuCyte® live cell analysis system (EssenBioScience) for cell proliferation analysis. All the experiments were repeated three times.

### Colony formation assay

ATF2 over-expressing or control cells were seeded in 12-well plates at 500cells/well. After four days, cells were treated with the indicated concentrations of cisplatin and cultured for another four days. Cells were washed with PBS, fixed with methanol for 20 min and stained with 0.1% crystal violet for 10 min. After washing with PBS, cell clones were visualized under a microscope. All experiments were repeated three times in triplicate.

### GST-Pull down assay

The TNT coupled transcription and translation system (Promega) was used to transcribe and translate Flag-p53 protein in vitro. Prokaryotically expressed GST-ATF2 in BL21 bacteria strain was purified with glutathione Sepharose 4B (GE Healthcare). Flag-p53 protein and GST-ATF2 were co-incubated and rotated in 1% NP-40 buffer at 4 ℃ for three hours. Subsequently, the protein complex was washed three times, eluted with 30 µl of 2 × SDS loading buffer and analyzed with Western Blotting assay.

### Immunohistochemistry

Paraffin-embedded sections of GC tissues were deparaffinized, rehydrated, and heated for antigen retrieval. After blocking with normal goat serum for 45 min at room temperature, the sections were incubated with ATF2 antibody (Santa Cruz Biotechnology) overnight at 4 °C. The EliVision plus kit and a 3’3-diaminobenzidine kit (MaiXin.Bio) were used to detect protein expression according to the manufacturers’ instructions. For H&E staining, sections were stained with hematoxylin and eosin (H&E) (Solarbio) according to the manufacturers’ instructions. The Medical Ethics and Human Clinical Trial Committee of Zhengzhou University approved the use of human GC tissues for research.

### Tumor xenografts

5 × 10^6^ of ATF2 over-expressing and control tumor cells were injected into 6-week-old IL2-rcg (IL2 receptor gamma chain) knock out immune-deficient male Syrian hamsters (Miao et al. in prepatation) via bilateral axillary (*n* = 5/group). When the average tumor volume reached 1500 mm^3^, the animals were treated with intraperitoneal (i.p.) injection of cisplatin (3 mg/kg) or PBS. Cisplatin was injected once weekly for a total of two times. Before animals presented cachexia, they were sacrificed and tumor samples were resected and fixed in formalin for subsequent immunohistochemistry. Hamster experiments were approved by the Animal Welfare and Research Ethics Committee of Zhengzhou University.

### RNA sequencing and real-time quantitative PCR

5 × 10^5^ HCT-116 or HCT-116 (p53^−/−^) cells that expressed vector or ATF2 were seeded into 6-well plates and cultivated overnight, then cells were treated with 4ug/ml cisplatin for 24 hours. Total RNA from cells were extracted by Trizol Reagent (Invitrogen, USA) and RNA sequencing assay was performed by GENEWIZ (Suzhou, China). For real-time quantitative PCR, 1ug total RNAs were reverse transcribed by PrimeScript™ RT Reagent kit (Takara, China) and the expression of target genes were detected with TB Green Premix Ex Taq™ II kit (Takara, China) using the following primers: GAPDH-F, 5’-CTGGGCTACACTGAGCACC-3’, GAPDH-R, 5’-AAGTGGTCGTTGAGGGCAATG-3’, CSK-F, 5’-CTGTACGCGCCTCATTAAACC-3’, CSK-R, 5’- CAGCATCACGTCTCCGAACTC-3’, NF1-F, 5’-CGAATCATCACCAATTCCGCA-3’, NF1-R, 5’- CCACAACCTTGCACTGCTTTAT-3’.

### Bioinformatics analysis

Protein-protein interaction was predicted by STRING software and the overlapping genes between ATF2 and p53 were predicted with Venny software, then KEGG pathway enrichment analysis of sharing genes were further analyzed using STRING. Discovery Studio software was used to perform protein docking analysis between crystal structure of ATF2 and p53. The genomic status of ATF2 and p53 in GC were analyzed by cBioPortal software. For RNA sequencing data, R studio software was used to identified differentially expression genes between vector and ATF2 overexpressing groups, then pathway enrichment of 2 fold elevated genes were analyzed with Metascape webtools, and heatmap was mapped using R studio.

### Statistical analysis

The overall survival was analyzed using Kaplan-Meier survival analysis and Log-rank test. Differences between the control groups and experiment groups were analyzed by one-way ANOVA and Student’s t test. All analysis was performed by SPSS version 21.0, and data were presented as mean ± SEM. *p* value of < 0.05 was considered statistically significant.

## Results

### ATF2 is associated with the survival of gastric cancer patients

**Fig. 1:**
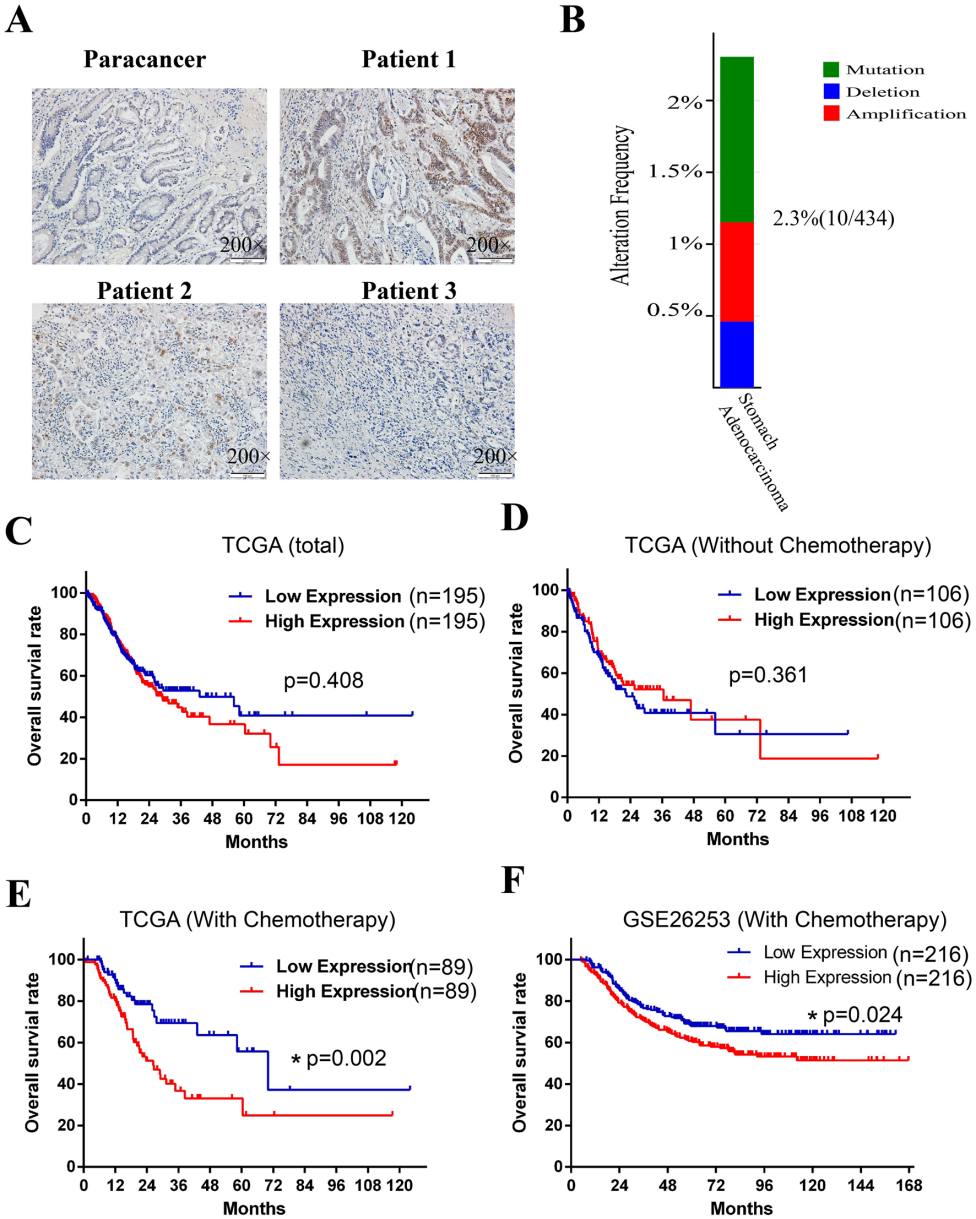
High expression of ATF2 in GC is associated with poor prognosis and chemotherapy response. **A** Immunohistochemistry staining was performed to evaluate the expression and location of ATF2 in human gastric cancer tissues. The original magnification is 200 ×. **B** Gene status of ATF2 including mutations (green), amplification (red), and deletions (blue) in GC tissue (n = 434) were analyzed using cBioportal software. **C** Overall survival of GC patients with ATF2-low or -high expression using TCGA database. **D** Overall survival of ATF2 low or -high expression group in GC patients without chemotherapy. **E** Overall survival of ATF2-low or ATF2-high expression group in GC patients with chemotherapy. **F** Overall survival of ATF2-low or ATF2-high expression group in GC patients with chemotherapy using GSE26253 dataset. Kaplan-Meier and Log-rank analysis is used, *p* value < 0.05 is considered statistically significant

To investigate the expression and location of ATF2 in human GC tissues, immunohistochemistry (IHC) assay was performed. As shown in Fig. 1 A, although ATF2 expression was elevated in some patients, most cancer cells exhibit low staining that similar with paracancer tissues. Besides, in that positive cells, ATF2 was mainly located in cellular nucleus. Subsequently, the gene status of ATF2 in GC was assessed with the cBioportal database. As shown in Fig. 1B, the frequency of gene mutation, amplification and deletion in GC tissues was rarely, indicating its expression was crucial for its function in tumor. To further explore the clinical significance of ATF2 expression in GC, the survival of GC patients with different ATF2 level was analyzed using TCGA database. Strikingly, data demonstrated that total patients or patients without chemotherapy exhibited no significant difference of the overall survival between the ATF2 high and ATF2 low groups (Fig. 1C, D), while high ATF2 group in patients that received chemotherapy had a poorer overall survival (Fig. 1E). This results was also validated by another GC GEO data (GSE26253) (Fig. 1F). All above data suggests that high expression of ATF2 was associated with poor prognosis and involved in a less effective chemotherapy response for GC.

### ATF2 is involved in cisplatin induced cell death

**Fig. 2:**
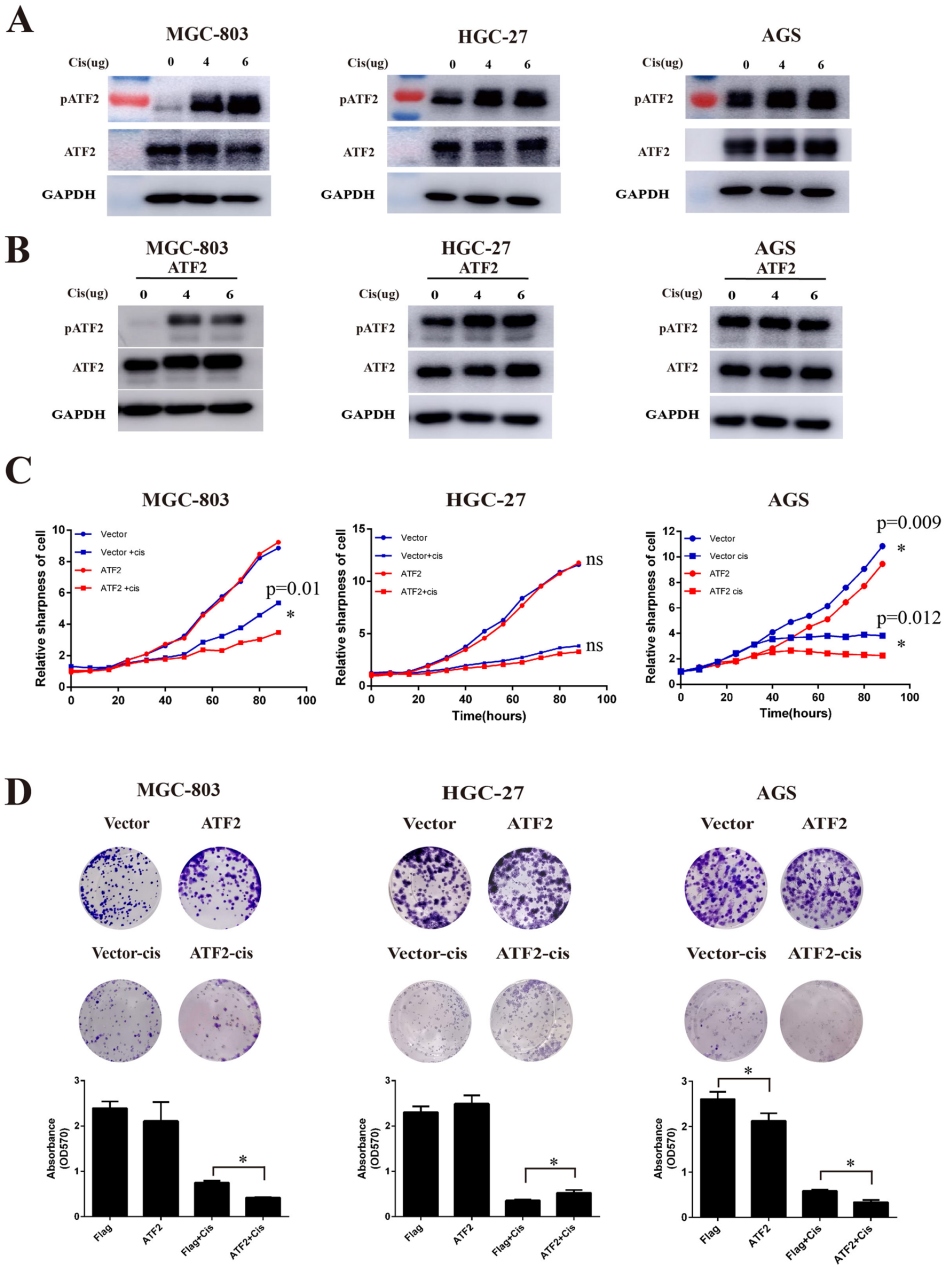
ATF2 is involved in cisplatin induced cell death. **A** Protein expression of ATF2 and phosphorylated-ATF2-Thr71 (p-ATF2) in different GC cell lines by Western Blotting after treatment with various doses of cisplatin. **B** Protein expression of ATF2 and p-ATF2 (phospho-Thr71) after treatment with different doses cisplatin in ATF2-overexpressing GC cell lines. **C** Cell proliferation and cytotoxicity of cisplatin (2 µg/ml) treated ATF2-overexpressing GC cell lines. **D** Cell colony formation ability of cisplatin (2 µg/ml) treated ATF2-overexpressing GC cell lines. Data are presented as mean ± SEM

Studies have shown that ATF2 is involved in disease development and DNA damage responses [17]. To further validate whether ATF2 is also plays a role in the response to chemotherapy, we firstly detected the expression of activated ATF2 (p-Thr71) in cisplatin treated GC cell lines. As shown in Fig. 2 A, the expression of p-ATF2 (phospho-Thr71) was increased with increasing doses of cisplatin in the GC cell lines MGC-803 and HGC-27, while total ATF2 expression did not change significantly. Interestingly, the expression of p-ATF2 in AGS cells was only slightly elevated. Similarly, results in ATF2 stable cell lines also showed that cisplatin treatment promoted the activation of ATF2 in MGC-803 and HGC-27 gastric cancer cells, while in AGS cells p-ATF2 changed little (Fig. 2B and Additional file 1: Fig.S1). Further cell proliferation and cytotoxicity assays demonstrated that ATF2 overexpression could promote cisplatin sensitivity in the AGS and MGC-803 cell lines, whereas no significant difference was observed in HGC-27 cell lines (Fig. 2 C). Colony formation assays showed that ATF2 could enhance cisplatin treatment induced cell death in MGC-803 and AGS cells, while promote cisplatin resistance in HGC-27 cells (Fig. 2D). These findings proved the effect of ATF2 on cisplatin induced cell death was dependent on cell types.

### p53 status affects ATF2 function

**Fig. 3:**
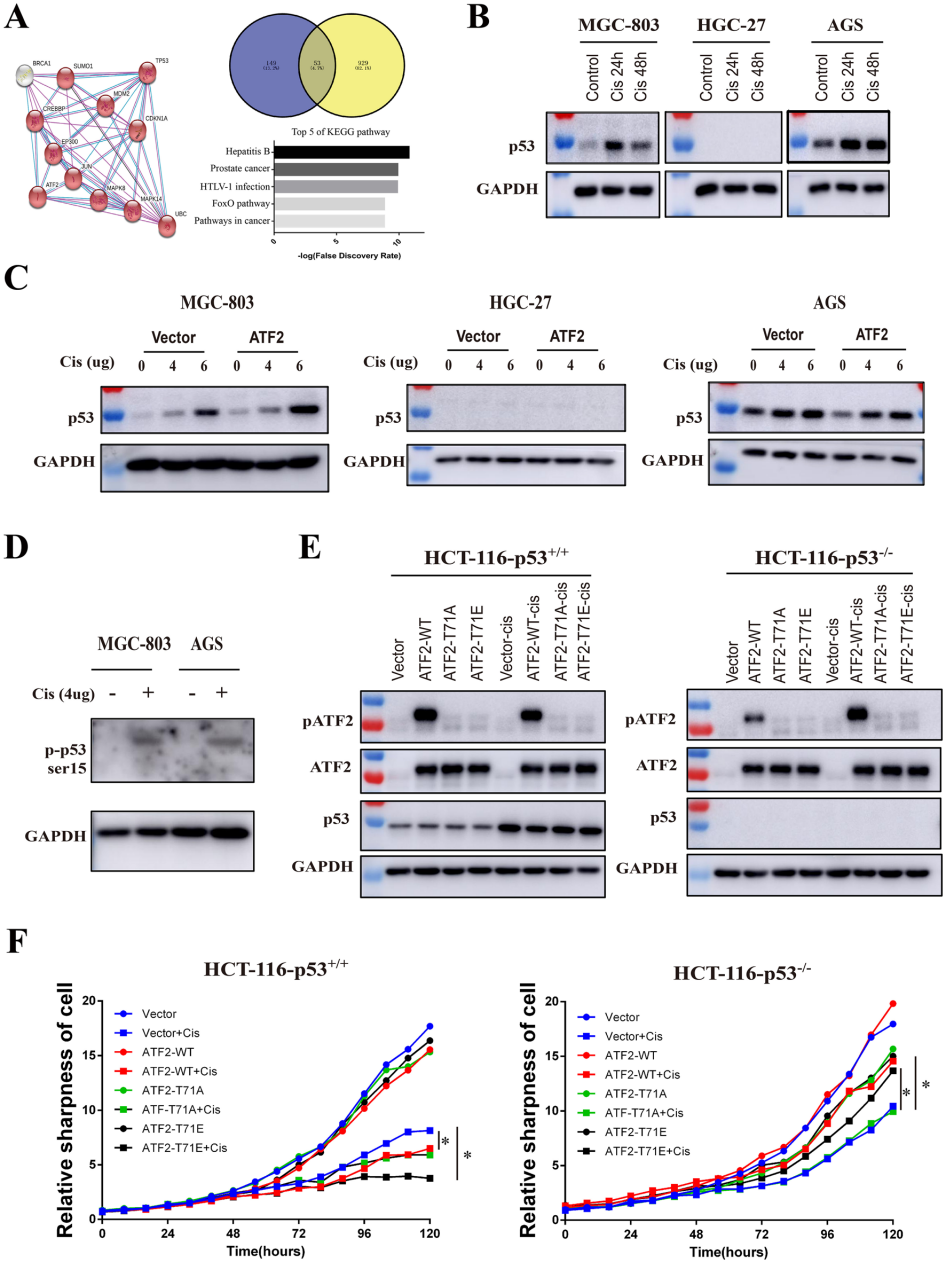
The genetic status of p53 affects the function of ATF2. **A** STRING database analysis was performed to explore potential interactions between ATF2 and p53, then the overlapped genes was predicted with Venny software, and the KEGG pathway enrichment of sharing genes were further analyzed with STRING, top 5 pathways were shown. **B** Protein expression of p53 in GC cells after cisplatin treatment for 24 or 48 h was analyzed by Western Blot assay. **C** Protein expression of p53 in GC cell lines treated with different doses of cisplatin was analyzed by Western Blot. **D** The phosphorylation of p53 in cisplatin treating MGC-803 and AGS were detected with Western Blot. **E** HCT116 p53+/+ or HCT-116 p53−/− cells that over-expressing different ATF2 subtypes (WT, T71A, T71E) were treated with cisplatin, then p53, ATF2 and p-ATF2 (phospho-Thr71) level were detected by Western Blot. ATF2 wild type (ATF2-WT), inactivated mutant type that mutated Thr to Ala (ATF2-T71A), activated mutant type that mutated Thr to Glu (ATF2-T71E). **F** Cell proliferation and cytotoxicity of cisplatin treated ATF2 subtype colorectal cancer cell lines with incucyte live cell analysis system. Cisplatin was used at a final concentration of 2 µg/ml

Abundant studies have demonstrated that p53 is associated with DNA damage and response to cancer chemotherapy. Given that MGC-803, HGC-27, and AGS cells have different p53 status (MGC-803 has site mutated p53/H179Q, HGC-27 has truncated p53/P153Afs*, AGS has wild type p53), we hypothesized that the gene status of p53 in cells may affect the function of ATF2. Protein interaction analysis with String software revealed both ATF2 and p53 bound with various molecules such as Jun, MAPK8, CREBBP, and EP300. Overlap analysis showed there were 53 binding partners shared by p53 and ATF2, moreover these overlapping genes were enrich in cancer pathway (Fig. 3A). Western blot demonstrated p53 protein level significantly increased in AGS and MGC-803 cells after cisplatin treatment, while no p53 was detected in HGC-27 cells (Fig. 3B). These results were also confirmed in ATF2 stable cell lines (Fig. 3C). Besides, cisplatin treatment could significantly increase p53 phosphorylation, which promotes its activation (Fig. 3D). To further study the role of ATF2 in cancer cells with different p53 status, HCT-116-p53^−/−^ and HCT-116-p53^+/+^ colon cancer cell lines over-expressing ATF2-WT (wild type), ATF2-T71A (inactivated), ATF2-T71E (activated) were treated with cisplatin. As shown in Fig. 3E, the level of activated ATF2 (p-ATF2) in HCT-116-p53^+/+^ overexpressing ATF2 (WT) cells exhibited not change after cisplatin treatment, while in HCT116-p53^−/−^ cells, cisplatin could significantly increase the phosphorylation of ATF2, which was consistent with the above findings in GC cells. To further confirm the effect of p53 on cisplatin sensitivity in ATF2-overexpressing colorectal cancer cell lines, cell proliferation assay was performed. As shown in Fig. 3F, in HCT-116-p53^+/+^ cells, both ATF2 and activated ATF2-T71E significantly enhanced cisplatin caused cell death. In contrast, in p53 deficient HCT116-p53^−/−^ cells, overexpressing ATF2 or activated ATF2-T71E showed more survival cells compared with control cells, demonstrating the gene status of p53 affected the function of ATF2 in GC.

### ATF2 interacts with p53 trans-activation domain

**Fig. 4:**
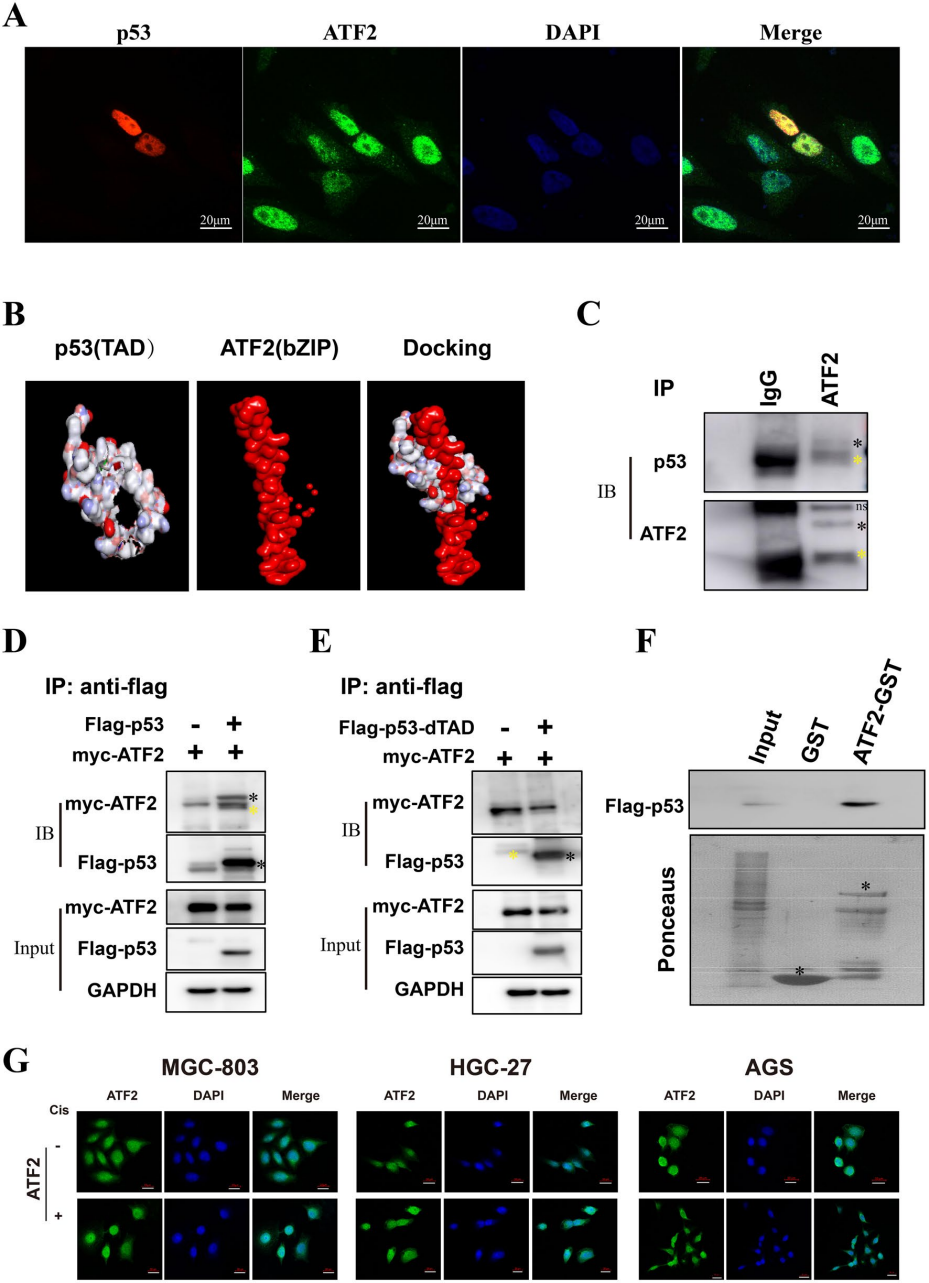
ATF2 interacts with the p53 TAD domain. **A** The co-localization of ATF2 (green) and p53 (red) GC cells was analyzed by confocal imaging. DAPI stains nuclear DNA (blue). The photomicrographs were taken at the original magnification 400 ×. **B** Molecular docking with the crystal structures of ATF2 and p53 were performed by Discovery Studio software. **C** The interaction between endogenous ATF2 and p53 was assessed with immunoprecipitation (IP) using ATF2 antibody. **D** Flag-p53 and Myc-ATF2 plasmids were co-transfected into cancer cells, then protein interaction was confirmed with immunoprecipitation (IP) assay. **E** Flag-p53-dTAD and Myc-ATF2 plasmids were co-transfected and an IP assay was performed. **F** The interaction between ATF2 and flag-p53 *in vitro* was elucidated using a GST-pull down assay. **G** The sub-cellular localization of ATF2 (green) in cisplatin treated GC cell lines was determined using immunofluorescence (400×). The positive band was marked with black star (*), and the heavy chain of antibody in IP was marked with yellow star (*)

To better understand the relationship between ATF2 and p53 in GC, we firstly investigated their location by immunofluorescence assay. As shown in Fig. 4A, both ATF2 and p53 were localized in the cell nucleus. Discovery Studio docking analysis indicated the bZIP domain of ATF2 could interact with p53 trans-activation domain (TAD) domain (Fig. 4B). To further investigate this hypothesis, immunoprecipitation (IP) and GST-pull down assay were conducted. As shown in Fig. 4 C, p53 could interact with ATF2 in cisplatin treating MGC-803 cells, which was further confirmed by co-transfection IP assay (Fig. 4D) and in-vitro GST-pull down assay (Fig. 4F). Moreover, the interaction disappeared when the TAD domain was deleted (dTAD) (Fig. 4E), indicating ATF2 interacted with p53 through the p53 TAD domain. As studies have previously demonstrated that stress stimuli affect ATF2 function by regulating its location, we investigated whether cisplatin treatment could affect the cellular sub-localization of ATF2 using immunofluorescence. Interestingly, cisplatin treatment promoted ATF2 nuclear export and translocation to the cytoplasm in p53 wild-type cells (AGS). However, there was no obvious change in p53 mutated cells (MGC-803 and HGC-27) (Fig. 4G).

### Regulation of ERK1/2 pathway by ATF2 is associated with p53 status

**Fig. 5:**
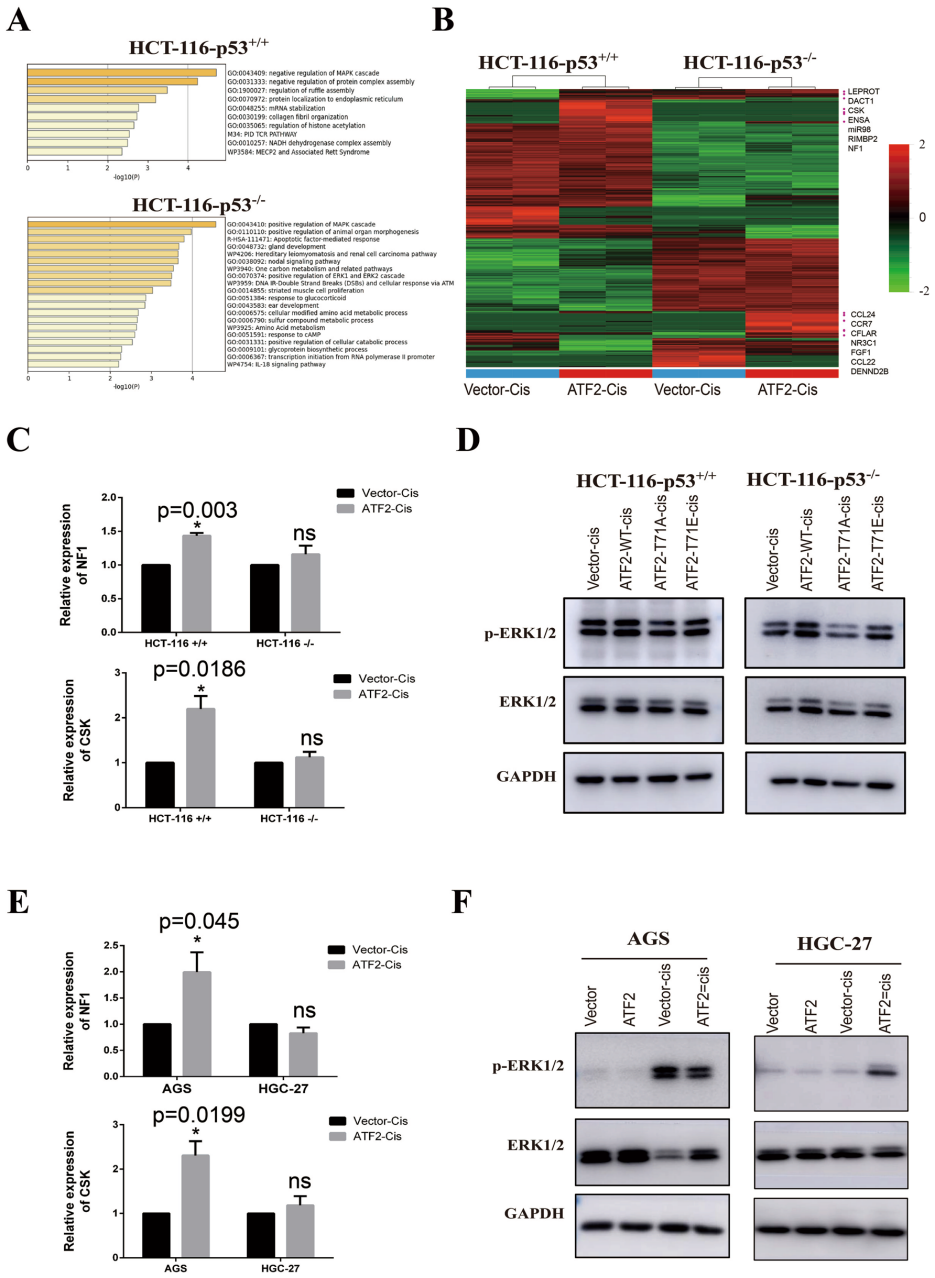
Regulation of ERK1/2 pathway by ATF2 is associated with p53. **A** Differential expressing genes between control and ATF2 overexpressing group in HCT-116 p53+/+ or HCT-116 p53−/− cell were identified with R studio, then gene oncology (GO) enrichment of elevated genes (foldchange > = 2) was analyzed with Metascape, and top GO biological processes were displayed. **B** Cluster analysis was performed using R studio and genes related with MAPK pathway were marked in the heatmap. **C** Relative expression of CSK and NF1 in cisplatin treated HCT-116 cells were measured with Real-time quantitative PCR assay. **D** Protein expression of total ERK1/2 and p-ERK1/2 (Thr202/Tyr204, activated) were detected with Western blot assay. **E** Real-time quantitative PCR assay was performed to evaluate the expression of CSK and NF1 in cisplatin treated GC cells. **F** Protein expression of total ERK1/2 and p-ERK1/2 (Thr202/Tyr204, activated) in GC cells were detected with Western blot assay

To clarify the two-faced role of ATF2 on cisplatin therapy, RNA sequencing was performed using paired ATF2 overexpressing HCT-116-p53−/− and HCT-116-p53+/+ cells. As shown in Fig. 5A, in p53 wild type HCT-116 cells, genes that upregulated by ATF2 were enriched in negative regulation of MAPK cascade, while in p53 deficient HCT-116-p53−/− cells, the elevated genes mainly enriched in positive regulation of MAPK cascade. As displayed in the Fig. 5B, MAPK negative regulators including LEPROT, CSK, NF1 were increasing in p53 wild type cells, while genes such as CCL24, FGF1 were elevated in p53 deficient cells. Considering several studied had proved that CSK and NF1 repressed ERK1/2 pathway, therefore we further validated sequencing results with Real-time quantitative PCR. As shown in Fig. 5C, overexpressing ATF2 promoted CSK and NF1 transcription in cisplatin treating HCT-116 cells, while had no significant effect in p53 deficient HCT-116 cells (Fig. 5C). Western blot proved ATF2 or ATF2-T71E was able to enhance the expression of activated ERK1/2 in HCT-116-p53−/− (Fig. 5D). These results were further confirmed in wild p53 AGS cells and truncated p53 HGC-27 cells (Fig. 5E, F). All these findings demonstrated the negative regulation of ERK1/2 pathway by ATF2 depends on p53 status.

### ATF2 was a chemotherapy resistance indicator for GC with dysfunctional p53

**Fig. 6:**
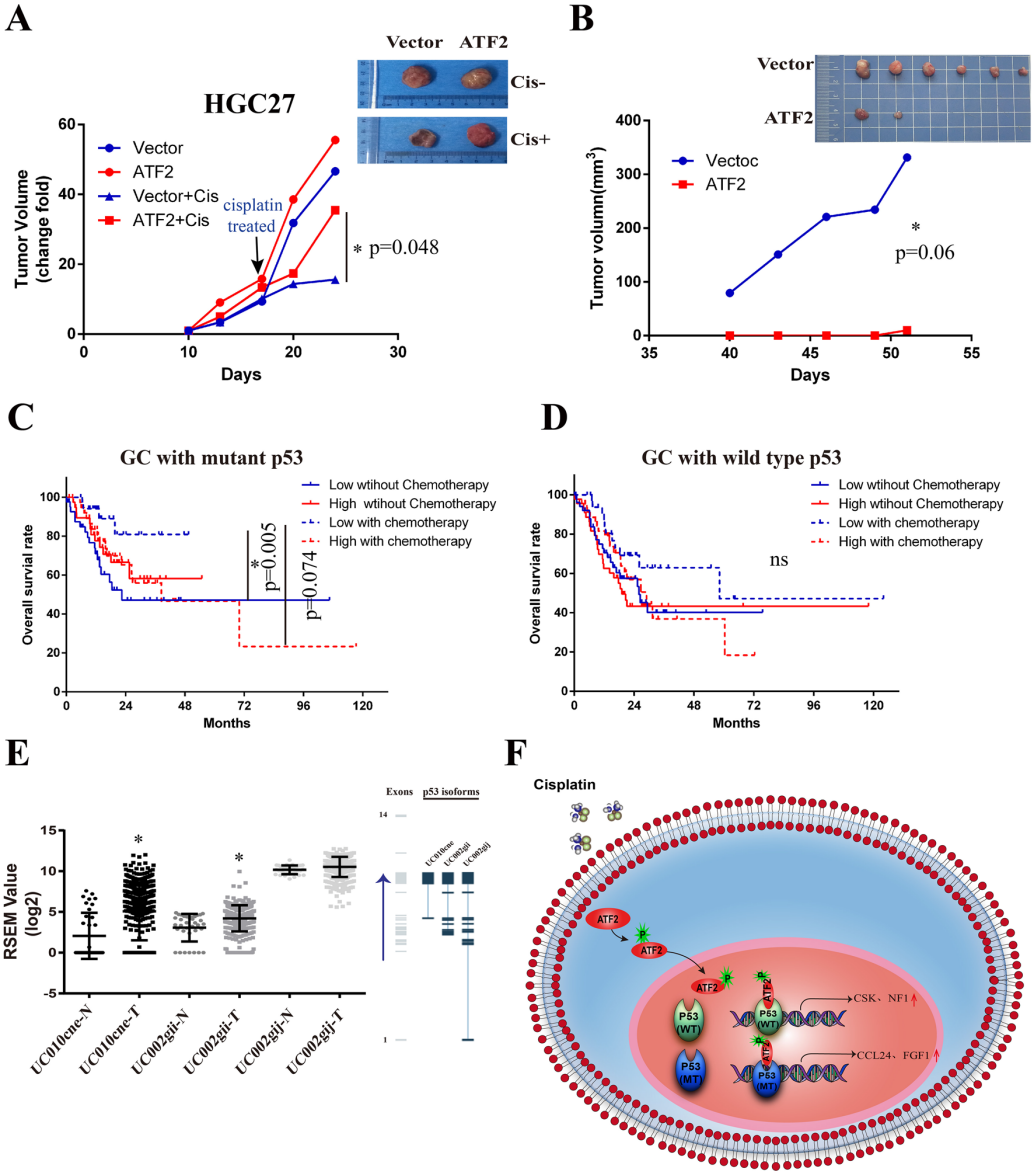
ATF2 was a chemotherapy resistance indicator for GC with dysfunctional p53. **A** Xenografts tumor size of ATF2 over-expressing group and control group with or without cisplatin treatment in HGC-27 cells. The animals were treated with intraperitoneal (i.p.) injection of cisplatin (3 mg/kg) or PBS when the average tumor volume reached 1500 mm^3^. Cisplatin was injected once weekly for two weeks. Data were presented as mean ± SEM (n = 5/group). **B** Xenografts tumor size of AGS over-expressing group and control group without cisplatin administration in AGS cells. **C** The overall survival of high and low ATF2 GC patients with or without chemotherapy when p53 was mutated. **D** The overall survival of high and low ATF2 GC patients with or without chemotherapy when p53 was wild type. **E** Statistical analysis of different p53 splicing variants in GC tissues using TCGA database. The right figure represents the number of exons contained in the p53 splicing variants, data provided by TSVdb database. UC010cne isoform lacked the 1st to 8th exons of p53; UC002-gii isoform lacked the1st to 4th exons of p53; UC002-gij isoform retains the full length of p53. N: normal adjacent noncancerous tissues. *T* tumor tissues. Paired-Samples t test is used, and *p < 0.05, **p < 0.01. **F** The model of the biological functions played by ATF2 in p53 mutated or wild type GC cells

To further determine the two-faced role of ATF2 in GC chemotherapy, ATF2 over-expressing HGC-27 and AGS were injected into immune-deficient Syrian hamsters (tumor formation ability is poor in nude mice, data not shown). For HGC-27 cells, there was no significant difference of tumor growth between control and ATF2 overexpressing groups without cisplatin administration, while the tumor size of ATF2 over-expressing group was larger than that of the control group after cisplatin treatment (Fig. 6A). Strikingly, the tumor growth of AGS cells were obviously inhibited in ATF2 overexpressing group even without cisplatin (Fig. 6B), indicating ATF2 was a tumor repressor in p53 wild AGS cancer cells. Subsequently, the overall survival of GC patients with different ATF2 expression and p53 status were further analyzed. As shown in Fig. 6C, in p53 mutation GC patients, lower expression ATF2 group with chemotherapy possessed better prognosis compared with un-treatment. Interestingly, in p53 wild type patients, no significantly difference was observed between all these four groups (Fig. 6D). Considering alternative splicing p53 which generate multiple isoforms were associated with tumor progress, so the alternative transcripts of p53 in GC were also analyzed. As shown in Fig. 6E, the alternative splicing rate of p53 that resulted in a lack of the N-terminal TAD domain in tumor tissues was significantly higher than found in adjacent noncancerous tissues, indicating p53 splice was also an influencer for ATF2 mediated GC chemotherapy. Taken together, our studies revealed that the effect of ATF2 on the cisplatin response of GC cells was associated with the genetic status of p53. Higher ATF2 expression could enhance cisplatin sensitivity in p53 wild type cell lines, while it promotes cisplatin resistance in p53 dysfunctional cancer cell lines (Fig. 6 F).

## Discussion

Activating transcription factor-2 (ATF2) is a transcriptional factor and sequence-specific DNA-binding protein with multiple roles in regulation of transcription of various genes, including those involved in anti-apoptosis, cell growth, DNA damage response, hypoxia [8, 17, 20–23]. In response to various stress stimuli or cytokines[24], ATF2 can be activated by kinases such as p38 MAPK, JNK, or ERK1/2 via phosphorylation of N-terminal residues Thr69 and Thr71, which conferred transcriptional activity to ATF2 [10, 11]. ATF2 can be also activated by Vaccinia-related kinase 1 (VRK1) through phosphorylation of Thr73 and Ser62 residues [12]. Study confirmed PKCε phosphorylates ATF2 at Thr52, which stimulates ATF2 nuclear localization, leading to a poorer survival prognosis of melanoma patients [25]. This study demonstrated that the chemotherapeutic drug cisplatin could promote the phosphorylation of ATF2 at Thr71, and that phosphorylation was associated with the genetic status of p53. We also found the location of ATF2 in cisplatin treated GC cells varied depending on the genetic status of p53.

Emerging evidence has confirmed that ATF2 can exert oncogenic or tumor suppressor activities depending on the cell or tissue in which it is expressed [26, 27]. For instance, ATF2 plays tumor-promoting roles in melanoma [21, 28, 29], non-small cell lung carcinoma [30] and pancreatic cancer cells [31], while it has tumor-suppressing activities in non-melanoma skin cancer [32, 33], breast cancer [10, 34], as well as in mouse orthotopic model of liver cancer [35]. Several studies concluded that the sub-cellular localization of ATF2 might be linked with human tumor stage and patient’s survival prognosis [4, 36, 37]. In addition, the opposing activities of ATF2 might be associated with its sub-cellular localization [28, 38, 39], ATF2 nuclear localization was associated with its oncogenic activity [16]. In this study, we found the subcellular localization of ATF2 was inconsistent with these observations in GC cells. In p53 wild-type AGS GC lines, cisplatin induced the activation of ATF2, promoting ATF2 nuclear export to cytoplasm. However, in p53 mutant or deficient cells, the localization of ATF2 remained nuclear after cisplatin treatment. Further experiments demonstrated the ATF2 was able to inhibited activation of ERK1/2 pathway by promoting NF1 and CSK1 expression in wild p53 cells, but enhanced ERK1/2 activation in p53 dysfunctional cells.

p53 which is mutated or inactivated in more than 50% human cancers, plays an essential role in maintaining genetic stability [40, 41]. Most p53 mutations are missense and located at the DNA-binding domain of p53, preventing p53 from transcribing its target genes and carrying out its normal function of preventing cancer formation [42]. Moreover, abundant studies have demonstrated different p53 isoforms are associated with tumor formation and adaptive stress response[43]. For the first time, this study proved ATF2 could interacted with the TAD domain of p53, and found p53 isoforms that lacks TAD domain is significantly increased in gastric cancer tissues.

In summary, this study proves the two-faced role of ATF2 on cisplatin response in GC is depend on p53 context. In p53 wild-type cells, ATF2 overexpression was able to promote cisplatin sensitivity, while enhanced cell survival in cisplatin treated p53 dysfunctional GC cells. ATF2 interacted with the TAD domain of p53 and exhibited opposite effect on ERK1/2 pathway in cells with different p53 status. In addition, patients with lower ATF2 and mutated p53 significantly benefit from chemotherapy. This study uncovers a novel molecular mechanism of ATF2 and provide new idea for precision treatment of GC.

## Conclusions

Our study demonstrates ATF2 could enhance tumor cisplatin sensitivity in p53 wild-type cancer cells, while promote chemotherapy resistance in cells with mutated p53. Further results reveals the regulation of ERK1/2 pathway by ATF2 is also depends on with p53 status. Integrated analysis of ATF2 expression and p53 status was potential indicator for chemotherapy sensitivity and prognosis of GC patients.

## Supplementary Information

**Additional file 1: Figure S1.** Protein expression of ATF2 in three cisplatin treated GC stable cell lines was analyzed by Western Blot.

## Data Availability

All the data and materials could be traced from the paper or can be requested from the corresponding author.

## Abbreviations

GC: Gastric cancer
ATF2: Activating transcription factor 2
bZIP: Basic leucine zipper
ERK1/2: Extracellular signal-regulated kinases1/2
TCGA: The cancer genome atlas (TCGA)
GEO: Gene Expression Omnibus
IP: Immunoprecipitation
PFA: Paraformaldehyde
TAD: Trans-activation domain
