# Supplementary figures and images for: The two-faced role of ATF2 on cisplatin response in gastric cancer depends on p53 context
Source: Cell Biosci. 2022 May 31;12:77. doi: 10.1186/s13578-022-00802-w (PMC9153165; doi:10.1186/s13578-022-00802-w)

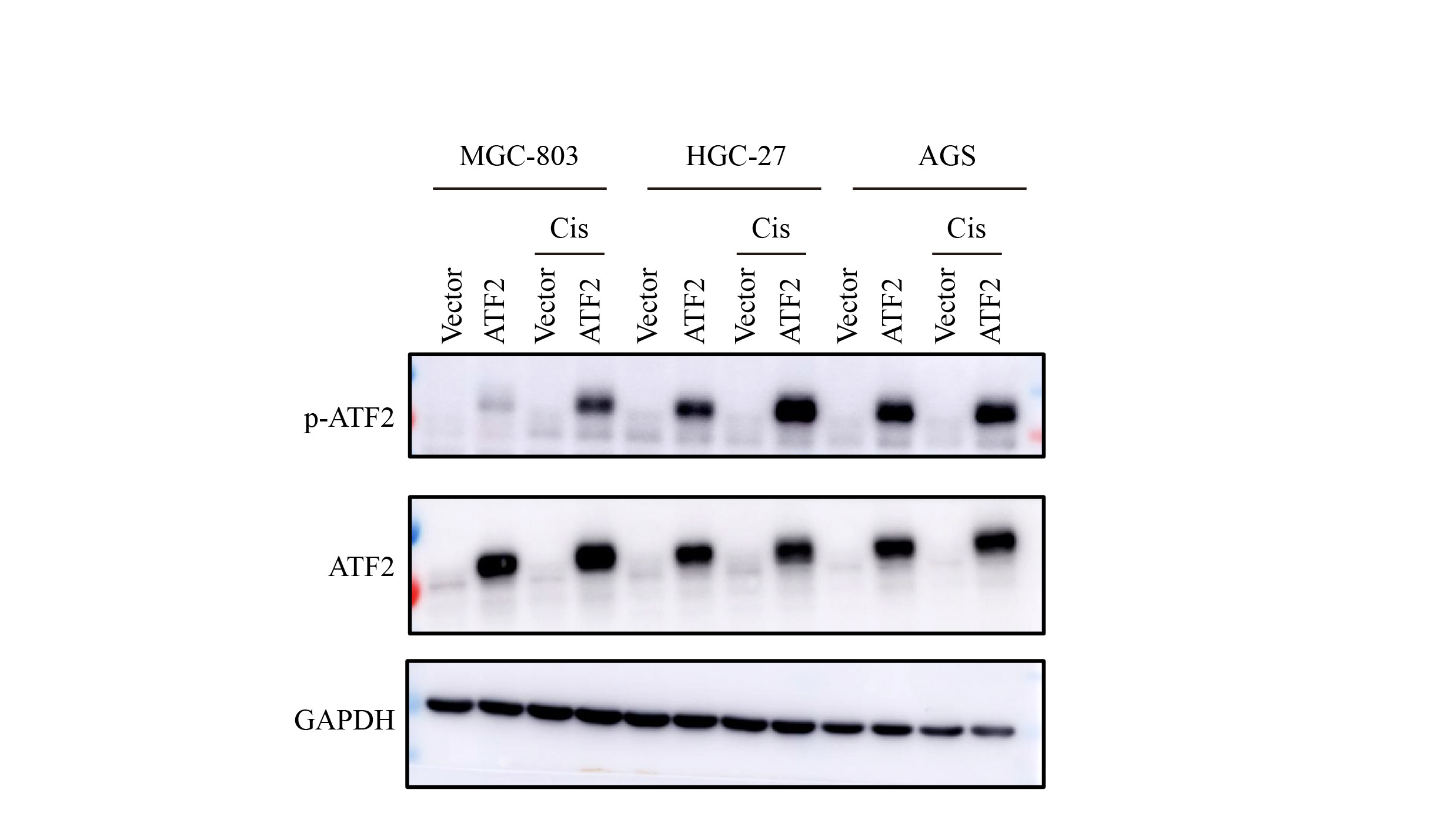

Supplement: Supplementary file 1 — Additional file 1: Figure S1. Protein expression of ATF2 in three cisplatin treated GC stable cell lines was analyzed by Western Blot. [file 13578_2022_802_MOESM1_ESM.jpg]
